# Ultra-High-Resolution Computed Tomography of the Lung: Image Quality of a Prototype Scanner

**DOI:** 10.1371/journal.pone.0137165

**Published:** 2015-09-09

**Authors:** Ryutaro Kakinuma, Noriyuki Moriyama, Yukio Muramatsu, Shiho Gomi, Masahiro Suzuki, Hirobumi Nagasawa, Masahiko Kusumoto, Tomohiko Aso, Yoshihisa Muramatsu, Takaaki Tsuchida, Koji Tsuta, Akiko Miyagi Maeshima, Naobumi Tochigi, Shun-ichi Watanabe, Naoki Sugihara, Shinsuke Tsukagoshi, Yasuo Saito, Masahiro Kazama, Kazuto Ashizawa, Kazuo Awai, Osamu Honda, Hiroyuki Ishikawa, Naoya Koizumi, Daisuke Komoto, Hiroshi Moriya, Seitaro Oda, Yasuji Oshiro, Masahiro Yanagawa, Noriyuki Tomiyama, Hisao Asamura

**Affiliations:** 1 Division of Cancer Screening, National Cancer Center, Research Center for Cancer Prevention and Screening, Chuo-ku, Tokyo, Japan; 2 Department of Radiology, National Cancer Center Hospital, Chuo-ku, Tokyo, Japan; 3 Department of Radiology, National Cancer Center Hospital East, Kashiwa, Chiba, Japan; 4 Department of Endoscopy, Respiratory Endoscopy Division, National Cancer Center Hospital, Chuo-ku, Tokyo, Japan; 5 Division of Pathology, National Cancer Center Hospital, Chuo-ku, Tokyo, Japan; 6 Department of Thoracic Surgery, National Cancer Center Hospital, Chuo-ku, Tokyo, Japan; 7 Department of CT Systems Division, Toshiba Medical Systems Corporation, Otawara, Tochigi, Japan; 8 Department of Clinical Oncology, Nagasaki University Graduate School of Biomedical Sciences, Nagasaki, Nagasaki, Japan; 9 Department of Diagnostic Radiology, Hiroshima University, Institute and Graduate School of Biomedical Sciences, Hiroshima, Hiroshima, Japan; 10 Department of Radiology, Osaka University Graduate School of Medicine, Suita, Osaka, Japan; 11 Department of Radiology, Niigata University Medical and Dental Hospital, Niigata, Niigata, Japan; 12 Department of Radiology, Niigata Cancer Center Hospital, Niigata, Niigata, Japan; 13 Department of Radiology, Ohara General Hospital, Fukushima, Fukushima, Japan; 14 Department of Diagnostic Radiology, Kumamoto University, Faculty of Life Sciences, Kumamoto, Kumamoto, Japan; 15 Department of Radiology, National Hospital Organization Okinawa National Hospital, Ginowan, Okinawa, Japan; Wayne State University, UNITED STATES

## Abstract

**Purpose:**

The image noise and image quality of a prototype ultra-high-resolution computed tomography (U-HRCT) scanner was evaluated and compared with those of conventional high-resolution CT (C-HRCT) scanners.

**Materials and Methods:**

This study was approved by the institutional review board. A U-HRCT scanner prototype with 0.25 mm x 4 rows and operating at 120 mAs was used. The C-HRCT images were obtained using a 0.5 mm x 16 or 0.5 mm x 64 detector-row CT scanner operating at 150 mAs. Images from both scanners were reconstructed at 0.1-mm intervals; the slice thickness was 0.25 mm for the U-HRCT scanner and 0.5 mm for the C-HRCT scanners. For both scanners, the display field of view was 80 mm. The image noise of each scanner was evaluated using a phantom. U-HRCT and C-HRCT images of 53 images selected from 37 lung nodules were then observed and graded using a 5-point score by 10 board-certified thoracic radiologists. The images were presented to the observers randomly and in a blinded manner.

**Results:**

The image noise for U-HRCT (100.87 ± 0.51 Hounsfield units [HU]) was greater than that for C-HRCT (40.41 ± 0.52 HU; *P* < .0001). The image quality of U-HRCT was graded as superior to that of C-HRCT (*P* < .0001) for all of the following parameters that were examined: margins of subsolid and solid nodules, edges of solid components and pulmonary vessels in subsolid nodules, air bronchograms, pleural indentations, margins of pulmonary vessels, edges of bronchi, and interlobar fissures.

**Conclusion:**

Despite a larger image noise, the prototype U-HRCT scanner had a significantly better image quality than the C-HRCT scanners.

## Introduction

High-resolution computed tomography (HRCT) of the lungs was first described in 1982; Todo et al. reported that HRCT improved the visualization of the fine structures of the lungs, such as the peripheral pulmonary vessels, terminal bronchioles, and interlobular septa [1]. Since then, HRCT of the lung has played an important role in the diagnosis of solitary pulmonary nodules [2–16]. However, the spatial resolution of conventional HRCT (C-HRCT) ranges from 0.23 [17] to 0.35 mm [18]. Recently, multislice CT and low-dose CT lung cancer screening have been used to detect numerous small indeterminate pulmonary nodules. To obtain more detailed images of solitary pulmonary nodules, we have developed a prototype ultra-high-resolution CT (U-HRCT) scanner.

The purpose of this study was to evaluate the image noise and image quality of a prototype U-HRCT scanner, compared with those of C-HRCT scanners.

## Materials and Methods

### Ethics statement

The National Cancer Center institutional review board approved this study. Written informed consent was obtained from all the patients.

### Specifications of the prototype U-HRCT scanner and the C-HRCT scanners

The prototype U-HRCT scanner (Toshiba Medical Systems, Otawara, Japan) is a 4-row detector CT scanner with a detector matrix size at the isocenter of 0.25 x 0.25 mm and a beam collimation of 0.25 mm x 4. The X-ray tube has a focus size of 0.6 x 0.6 mm and a maximum output of 120 kV and 160 mA or 135 kV and 140 mA; the milliamperes second (mAs product) is 120 or 105, respectively, with a gantry rotation time of 0.75 seconds per rotation. The maximum scanning field of view (SFOV) is 250 mm, and the maximum scanning time is 30 seconds. The bore size is 72 cm.

One of the C-HRCT scanners is a 16-row detector CT scanner (Aquilion 16; Toshiba Medical Systems, Otawara, Japan) with a detector matrix size at the isocenter of 0.5 x 0.5 mm and a beam collimation of 0.5 mm x 16. The X-ray tube has a focus size of 0.9 x 0.8 mm and a maximum output of 120 kV and 500 mA. The gantry rotation time is 0.5 seconds per rotation. The maximum SFOV is 500 mm. The other C-HRCT scanner is a 64-row detector CT scanner (Aquilion 64; Toshiba Medical Systems, Otawara, Japan); the specifications were the same for the detector matrix size, focus size, maximum output, gantry rotation speed, and maximum SFOV, but the collimation is 0.5 mm x 64. In both C-HRCT scanners, the bore size is 72 cm; the spatial resolution is 0.35 mm, and the contrast resolution is 2.5 mm (0.25%).

**Fig 1:**
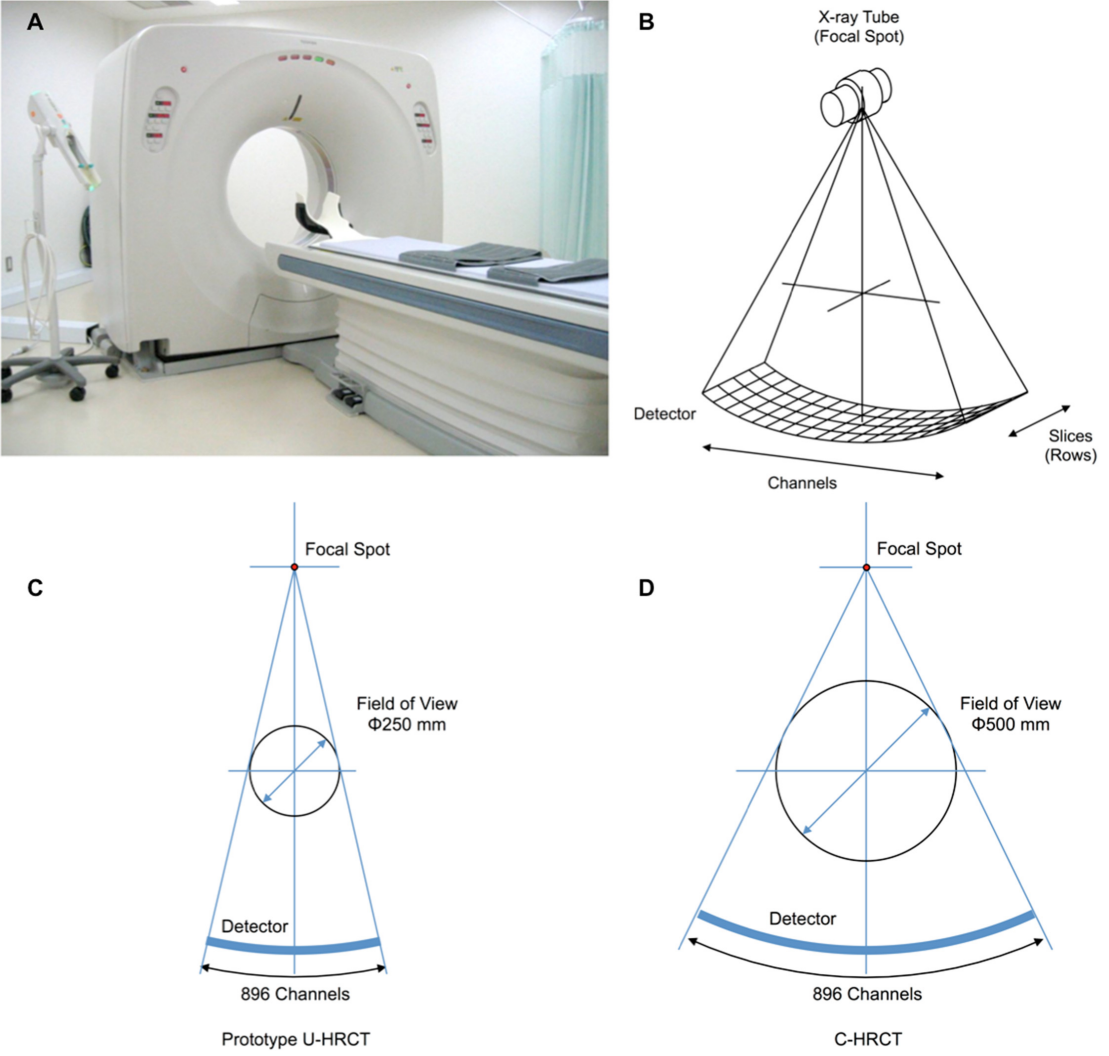
Appearance and geometry of channel direction of the prototype ultra-high-resolution CT scanner. (A) External appearance of the prototype ultra-high-resolution CT scanner. The bore size was 72 cm in diameter. (B) Image showing the directions of the slices and the channels of the CT detector. (C) Geometry of channel direction of the prototype ultra-high-resolution CT scanner. The maximal field of view was 250 mm and number of the channels was 896. (D) Geometry of channel direction of the conventional high-resolution CT scanner. The maximal field of view was 500 mm and number of the channels was 896. U-HRCT: ultra-high-resolution CT, C-HRCT: conventional high-resolution CT.

An overview of the prototype U-HRCT scanner is shown in Fig 1A. Fig 1B shows the directions of slices and channels of the CT detector, and Fig 1C and 1D show the relations among the focal spot (X-ray tube), the maximal field of view of the scan, and the detector for the prototype U-HRCT and the C-HRCT scanners.

### Protocols for U-HRCT and C-HRCT scanning

The pitch factor of the U-HRCT scanner was 0.625 (volume CT dose index CTDI_vol_ = 36.6 mGy [120 kV, 120 mAs], 39.1 mGy [135 kV, 105 mAs]), and the images were reconstructed using 0.25-mm-thick slices at 0.1-mm intervals.

Between December 2008 and June 2010, the scanning parameters for C-HRCT using the 16-row multislice CT scanner were 120 kV, 300 mA, a beam collimation of 0.5 mm x 16, a gantry rotation speed of 0.5 seconds per rotation, and a pitch factor of 0.688 (CTDI_vol_ = 34.6 mGy [120 kV, 150 mAs]). After July 2010, the scanning parameters for C-HRCT using the 64-row multislice CT scanner were the same for kV, mA, gantry rotation speed, pitch factor, and CTDI_vol_ but a beam collimation was 0.5 mm x 64. C-HRCT images were reconstructed using 0.5-mm-thick slices at 0.1-mm intervals.

Both U-HRCT and C-HRCT images were reconstructed using high-resolution lung algorithms and an 80-mm display FOV (DFOV). The lung window settings for U-HRCT and C-HRCT were a width of 1600 Hounsfield units (HU) and a level of –600 HU.

For U-HRCT, the scanning procedure was as follows: first, when a pulmonary nodule was located in the right lung of the patient, he or she was placed in a dorsal position on the bed so that the right-hand side of the back occupied the center of the bed; second, a scanogram was obtained; third, low-dose scanning (CTDI_vol_ = 3.0 mGy [120 kV, 22 mAs]) was performed to confirm the location of the nodule with a coverage of a 10-cm range of the thorax, but the whole thorax was not scanned; finally, standard- dose scanning was performed with a 2-cm range of coverage of the thorax within which the pulmonary nodule had been visualized. For C-HRCT, scanning was performed within a 4-cm range of the thorax in which a pulmonary nodule had been located after the whole thorax had been scanned using a low-dose setting (CTDI_vol_ = 2.2 mGy [120 kV, 15 mAs]). For both U-HRCT and C-HRCT, breath-holds were performed at full inspiration; intravenous contrast media was not used. The median interval between the U-HRCT scan and C-HRCT scan was 0 days (range, 0–35 days; lower quartile, 0; upper quartile, 2).

### Physical evaluations

#### Image noise

Image noise for the prototype U-HRCT and C-HRCT images (Aquilion 64) was evaluated using a 240-mm-diameter cylindrical water phantom. Scanning was performed 20 times with each scanner using the same protocol and reconstruction settings as described above. Image noise was evaluated based on the mean standard deviation of the CT values of the phantom in each CT image according to the International Electrotechnical Commission (IEC) standard [18].

#### Spatial resolution

**Fig 2:**
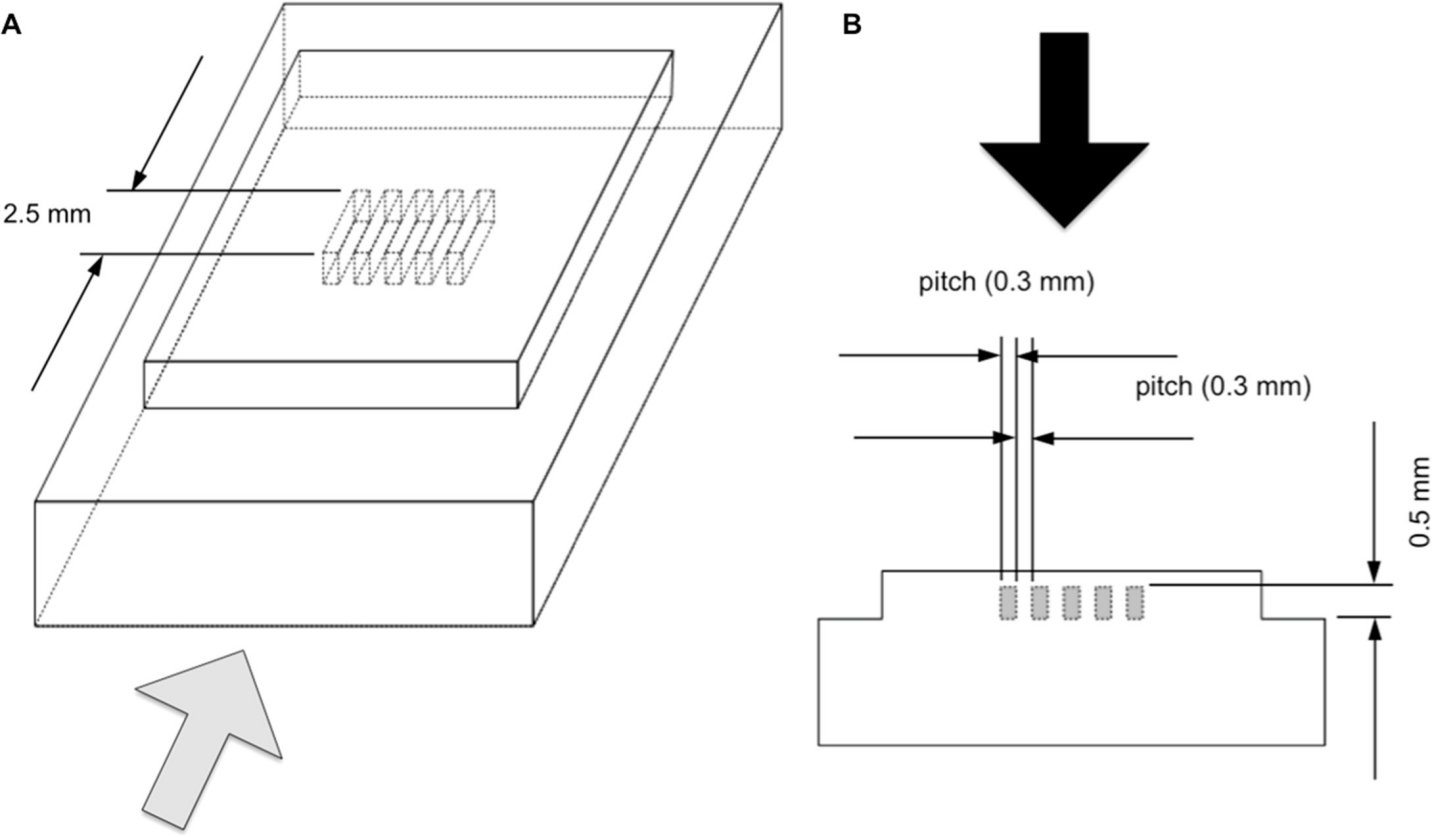
Schematic diagram of a slit phantom. (A) Bird’s-eye view of the slit phantom. The phantom consists of two rectangular parallelepipeds. A small rectangular parallelepiped with five slits was fixed on a large rectangular parallelepiped. (B) Lateral view of the slit phantom. The gray rectangles on the lateral view of the slit phantom (the side of the phantom as viewed from the gray arrow in Fig 2A) indicate the slits; the space between the slits is the same as the width of the slits. The slit phantom was photographed under a microscope; the black arrow indicates the angle at which the photographs were taken.

The spatial resolution of the U-HRCT images and the C-HRCT images (Aquilion 64) was evaluated using acrylic resin slit phantoms specifically developed for this study (Fig 2A). The slits and the intervening spaces were the same width in each phantom (Fig 2B). Each phantom was set up based on the rotation of a gantry and was scanned using the protocols for each scanner. For U-HRCT, the scanning and reconstruction protocols were as follows: 120 kVp, 160 mA, a beam collimation of 0.25 mm x 4, 0.75 seconds per rotation, and a conventional scan setting. The images were reconstructed using 0.25-mm-thick slices at 0.1-mm intervals. For C-HRCT, the scanning and reconstruction protocols were as follows: 120 kVp, 160 mA, a beam collimation of 0.5 mm x 4, 0.75 seconds per rotation, and a conventional scan setting. The images were reconstructed using 0.5-mm-thick slices at 0.1-mm intervals. Both the U-HRCT and C-HRCT images were reconstructed using high-resolution lung algorithms and an 80-mm DFOV.

### Observer test for evaluating image quality

Experienced observers evaluated the quality of diagnostically relevant findings on the U-HRCT and C-HRCT images of lung nodules that had been detected using low-dose CT lung cancer screening.

#### Patients

From December 2008 through May 2011, 44 nodules in 42 patients were examined using both U-HRCT and C-HRCT. Twenty-eight patients were scanned on the 16-row C-HRCT scanner and 14 patients on the 64-row C-HRCT scanner. All the nodules had been detected during low-dose CT screening for lung cancer at the Research Center for Cancer Prevention and Screening, and both U-HRCT and C-HRCT were performed as part of the follow-up care. As a training session before the observer test, 8 images selected from 8 nodules in 7 patients (1 woman and 6 men; mean age, 72.8 years [range, 70–78 years]) were selected. The nodules had a mean maximal diameter of 10.2 mm (range, 6.2–14.5 mm). The consistency was part-solid (lesions consisted of both solid soft-tissue attenuation, which obscures the bronchial and vascular margins, and ground-glass attenuation [i.e., hazy increased attenuation], which does not) [19] for 3 nodules and solid for 5 nodules. The diagnoses were nodular pneumonia in 2 cases, benign nodules in 2 cases (no change in the size or shape after more than 2 years of follow-up), and invasive adenocarcinoma (lepidic-predominant), mucosa-associated lymphoid tissue (MALT) lymphoma, ciliated muconodular papillary tumor, and suspected adenocarcinoma requiring follow-up in 1 case each. Fifty-three images selected from 37 nodules from 36 patients (24 women and 12 men [1 man overlaps 1 patient in the training session]); mean age, 63.2 years [range, 42−80 years]) were selected for use in the observer test. Several different images showing different CT findings were selected from among the total number of nodules that were examined as follows: 3 images of a single nodule, 2 images from each of 14 nodules, and 1 image from each of 22 nodules were selected. Thus, 53 images were selected from a total of 37 nodules. The mean maximal diameter of these nodules was 12 mm (range, 8−18 mm); the consistency was pure ground glass for 1 nodule, part-solid for 28 nodules, and solid for 8 nodules. Of the 37 nodules, 25 nodules had been resected as of February 2011, and 22 had been diagnosed as adenocarcinomas (6 adenocarcinomas in situ, 13 minimally invasive adenocarcinomas, 3 invasive adenocarcinomas [2 lepidic-predominant and 1 acinar-predominant]), 1 intrapulmonary lymph node, 1 MALT lymphoma (another image from the case of MALT lymphoma selected for the training session), and 1 case of histoplasmosis. Of the remaining 12 nodules, which had not been resected, 5 nodules in 5 patients are still being followed up because no definitive growth or increase in density has been observed, despite a suspicion of adenocarcinoma, and 7 nodules were thought to be benign because no changes in their size or shape have been observed after more than 2 years of follow up. The CT images were stripped of all patient information and were assigned anonymous identification numbers for the observer test.

#### Reading procedure

**Fig 3:**
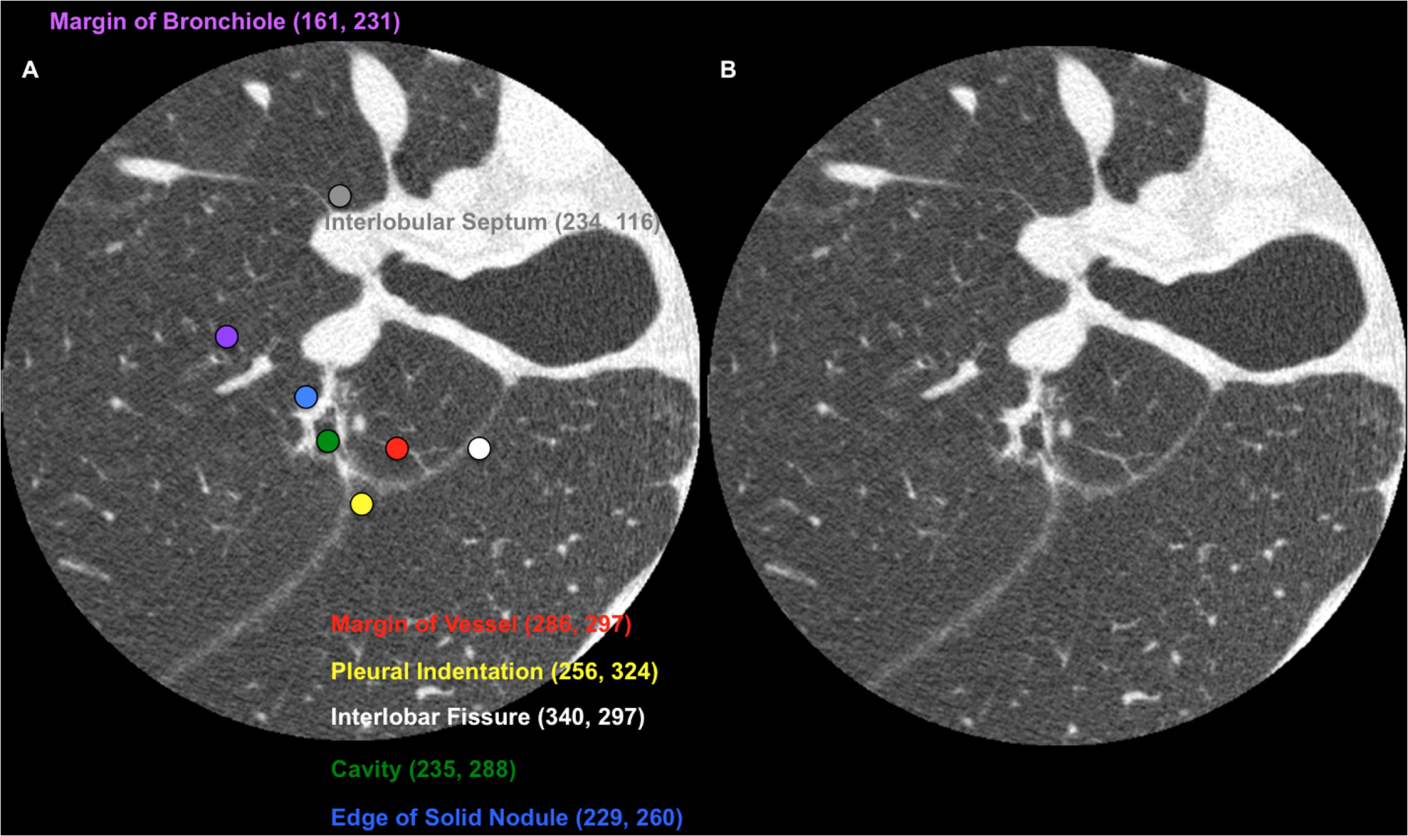
Example of CT images for the observer test. (A) An example of an ultra-high-resolution CT image with different colored dots and annotations used for the observer test. The X and Y coordinates of each dot are shown in parentheses. The image shows a lepidic-predominant invasive adenocarcinoma with a tumor size of 1.2 x 0.9 x 0.9 cm located in segment 2 of the right upper lobe in a 73-year-old man. The blue dot indicates the edge of a solid nodule. The green dot indicates a cavity. The yellow dot indicates pleural indentation of the interlobar fissure. The white dot indicates a fissure between the right upper lobe and the right lower lobe. The red dot indicates the margin of the pulmonary vessel. The purple dot indicates the edge of a bronchiole. The gray dot indicates an interlobular septum. (B) Ultra-high-resolution CT image in which the dots and annotations are hidden.

The reading system consisted of a server and 2 computers, each with 2 monitors (a 1.3-megapixel liquid crystal color display monitor for data input and a 9-megapixel liquid crystal color display monitor for viewing). The participants in the observer test were 10 board-certified thoracic radiologists who had 7 to 30 years (mean, 14 years) of experience in reading CTs. As they viewed the CT images, the observers were asked to evaluate the quality of the CT findings and assign scores. First, the observers underwent a training session using images of 8 nodules: each of a U-HRCT image and a corresponding C-HRCT image of the same nodule were presented sequentially in a nonblinded manner. After the training session, the observer test was performed with 53 images selected from the 37 nodules being presented to the observers: each of the U-HRCT image and a corresponding C-HRCT image of the same nodule were presented randomly in a blinded manner. The CT findings to be scored were as follows: margins of subsolid and solid nodules, the edges of solid components, pulmonary vessels in subsolid nodules, air bronchograms, pleural indentations, margins of pulmonary vessels, edges of bronchi and bronchioles, interlobar fissures, and interlobular septa. On each CT image, the CT findings to be scored were indicated using differently colored dots. Each dot was annotated with its X and Y coordinates so that the observers could identify the position of each CT finding on the CT image (Fig 3A). The observers were able to hide (Fig 3B) or show (Fig 3A) these dots and annotations simultaneously on the viewer to evaluate and score each CT finding.

#### Subjective evaluation using a 5-point score of image quality

Each CT finding was subjectively evaluated and graded using a 5-point score of image quality as follows: “1” indicated the worst image quality (no findings detectable), “2” indicated poor image quality (findings can be detected but the margin or internal characteristics are difficult to evaluate), “3” indicated fair image quality (partially indistinct findings can be detected and the margin or internal characteristics can be evaluated), “4” indicated good image quality (some indistinct findings can be detected and the margin or internal characteristics can be evaluated), and “5” indicated excellent image quality (findings are extremely clear and easy to detect and the margin or internal characteristics can be evaluated) [20]; i.e., “1” was the worst possible score and “5” was the best (S1 Table).

The identification number for each observer was irreversibly anonymized before the data were analyzed.

#### Interobserver agreement

The interobserver agreement for the 5-point score described above was evaluated using κ statistics. The κ values were regarded as follows: 0.00–0.20 was poor, 0.21–0.40 was fair, 0.41–0.60 was moderate, 0.61–0.80 was good, and 0.81–1.00 was excellent; the higher the κ values, the stronger in agreement between two observers.

### Representative CT-pathologic correlation of a part-solid nodule

In order to correlate the three sets of figures, i.e., sections of the histopathological specimen, the U-HRCT images, and the C-HRCT images, three-dimensional (3D) multiplanar reconstruction (MPR) of each set of CT data was performed using a commercially available image processing workstation (Ziostation; Ziosoft Inc., Tokyo, Japan). Maximum intensity projection (MIP) images of the pulmonary vessels and 3D curved-MPR of the pulmonary bronchi were also obtained from each set of CT data.

### Statistical analyses

The Mann-Whitney U test and Wilcoxon signed-rank test were performed using the JMP version 9 software program (SAS Institute, Cary, NC, USA) and the κ statistics were determined using MedCalc statistical software, version 13 (MedCalc Software bvba, Ostend, Belgium). The Mann-Whitney U test was used to evaluate image noise, and the Wilcoxon signed-rank test was used to evaluate the image-quality scores of the U-HRCT and C-HRCT findings. A *P* value <0.05 was considered significant.

## Results

### Assessment of image noise and spatial resolution

**Fig 4:**
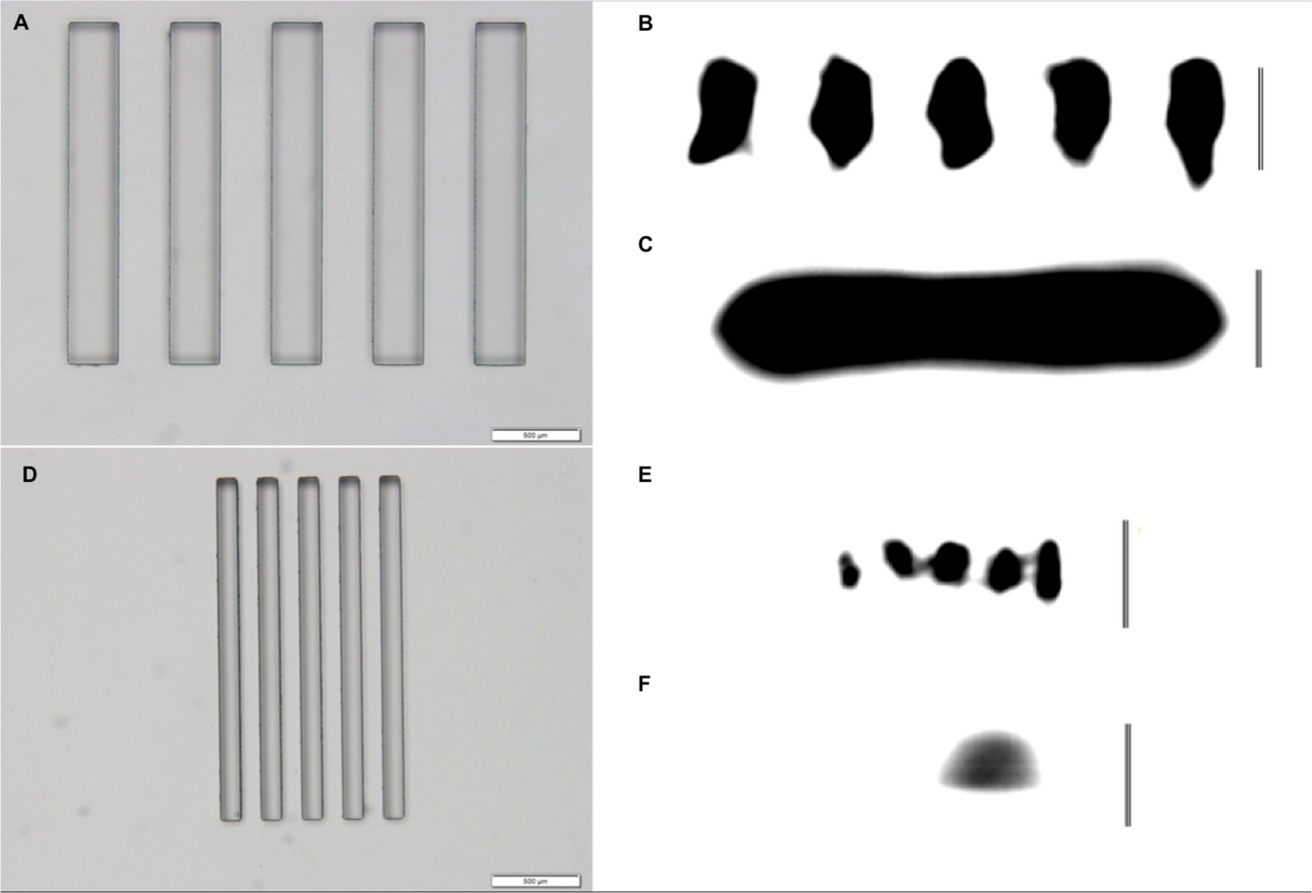
Loupe view of the slit phantoms and the results of CT scan. (A) Loupe view of the 0.3-mm slit phantom as viewed from above. The scale at the right lower part of the figure shows a distance of 0.5 mm. (B) Ultra high-resolution CT was capable of depicting a 0.3-mm slit. The vertical bar at the right side of the figure indicates a distance of 0.5 mm. (C) Conventional high-resolution CT was incapable of depicting the 0.3-mm slit. The vertical bar at the right side of the figure indicates a distance of 0.5 mm. (D) Loupe view of the 0.12-mm slit phantom as viewed from above. The scale in the right lower part of the figure indicates a distance of 0.5 mm. (E) Ultra high-resolution CT was capable of depicting a 0.12-mm slit. The vertical bar at the right side of the figure indicates a distance of 0.5 mm. (F) Conventional high-resolution CT was incapable of depicting the 0.12-mm slit. The vertical bar at the right side of the figure indicates a distance of 0.5 mm.

The image noise for U-HRCT (100.87 ± 0.51 HU, 0.25 mm-slice) was more than twice as great as that for C-HRCT (40.41 ± 0.52 HU, 0.5 mm-slice) (*P* < .0001). However, U-HRCT was capable of depicting a 0.12-mm slit, whereas C-HRCT was incapable of depicting even a 0.3-mm slit (Fig 4).

### Evaluation of image quality

The image quality of all the U-HRCT findings was scored higher than that of all the 16-row C-HRCT findings (*P* < .0001) (Table 1). The image quality of all the U-HRCT findings but 2 (margin of bronchiole and interlobular septa) was scored higher than that of the 64-row C-HRCT findings (*P* < .0001) (Table 2); although both the means of scores in margin of bronchioles and interlobular septa on the U-HRCT findings were higher than those on the 64-row C-HRCT findings, differences were not statistically significant because of small number of sample size. Some pairs of CT findings were not scored because of the not-user-friendly interface of the reading system.

**Table 1:** Comparison of Scores for U-HRCT and 16-row C-HRCT Findings. U-HRCT: ultra-high-resolution CT, C-HRCT: conventional high-resolution CT.

Findings on CT images	Scores of U-HRCT findings(mean ± SD)§	Scores of C-HRCT findings(mean ± SD)§	*P* value	Number of pairs of CT findings evaluated by the observers	Number of pairs of CT findings not scored by the observers	Number of pairs of CT findings included in the evaluation
**Margin of pulmonary vessels**	4.0 ± 0.9	3.3 ± 0.8	< .0001	329	1	330
**Margin of bronchi**	3.6 ± 1.0	3.0 ± 0.8	< .0001	214	6	220
**Margin of bronchioles**	3.5 ± 1.0	2.0 ± 0.7	< .0001	60	0	60
**Air bronchograms**	3.8 ± 0.9	3.0 ± 0.9	< .0001	216	4	220
**Edge of ground-glass opacities**	3.1 ± 0.9	2.8 ± 0.8	< .0001	189	1	190
**Edge of solid components in part-solid nodules**	3.8 ± 0.9	3.1 ± 0.8	< .0001	128	2	130
**Interlobular septa**	3.1 ± 1.0	2.5 ± 0.8	< .0001	96	4	100
**Edge of vessels in ground-glass opacities**	3.6 ± 1.0	2.9 ± 0.7	< .0001	80	0	80
**Edge of solid nodules**	3.8 ± 0.9	3.4 ± 0.9	< .0001	119	1	120
**Interlobar fissures**	3.6 ± 0.7	2.9 ± 0.6	< .0001	103	7	110
**Pleural indentations**	3.5 ± 0.7	2.9 ± 0.7	< .0001	70	0	70

§: Score was rounded to the first decimal place.

**Table 2:** Comparison of Scores for U-HRCT and 64-row C-HRCT Findings. U-HRCT: ultra-high-resolution CT, C-HRCT: conventional high-resolution CT.

Findings on CT images	Scores of U-HRCT findings(mean ± SD)§	Scores of C-HRCT findings(mean ± SD)§	*P* value	Number of pairs of CT findings evaluated by the observers	Number of pairs of CT findings not scored by the observers	Number of pairs of CT findings included in the evaluation
**Margin of pulmonary vessels**	4.2 ± 0.9	3.3 ± 0.8	< .0001	200	0	200
**Margin of bronchi**	3.9 ± 1.0	3.1 ± 0.9	< .0001	167	3	170
**Margin of bronchioles**	3.2± 1.0	2.8± 1.0	0.0938	20	0	20
**Air bronchograms**	3.8 ± 1.0	2.7 ± 0.9	< .0001	150	0	150
**Edge of ground-glass opacities**	3.5 ± 0.9	2.9 ± 0.7	< .0001	179	1	180
**Edge of solid components in part-solid nodules**	3.8 ± 0.9	3.0 ± 0.7	< .0001	129	1	130
**Interlobular septa**	3.4 ± 1.2	3.0 ± 0.7	0.2183	20	0	20
**Edge of vessels in ground-glass opacities**	3.6 ± 1.1	2.5 ± 0.8	< .0001	79	1	80
**Edge of solid nodules**	4.2 ± 0.9	3.5 ± 0.9	< .0001	30	0	30
**Interlobar fissures**	3.8 ± 0.8	2.7 ± 0.6	< .0001	59	1	60
**Pleural indentations**	3.9 ± 0.6	2.9 ± 1.0	< .0001	30	0	30

§: Score was rounded to the first decimal place.

### Interobserver agreement

The interobserver agreement is shown in Table 3. When the 10 observers were compared in pairs of 2 observers, 45 combinations of paired observes were possible. Although the maximal κ value for each CT finding made using each CT scanner ranged from moderate to excellent, the mean κ value derived from the κ values of any 2 observers (45 possible combinations of paired observers) for each CT finding made using each CT scanner ranged from poor to fair.

**Table 3:** Interobserver Agreement for U-HRCT and C-HRCT Finding. U-HRCT: ultra-high-resolution CT, C-HRCT: conventional high-resolution CT.

			κ Value			
	Mean		Minimum		Maximum	
Findings on CT images	U-HRCT	C-HRCT	U-HRCT	C-HRCT	U-HRCT	C-HRCT
**Margin of pulmonary vessels**	0.142	0.071	-0.069	-0.024	0.745	0.541
**Margin of bronchi**	0.244	0.194	-0.023	-0.138	0.633	0.628
**Margin of bronchioles**	0.071	0.253	-0.2	0	0.6	0.68
**Air bronchograms**	0.277	0.251	0.02	0.006	0.594	0.605
**Edge of ground-glass opacities**	0.252	0.321	0.022	0.012	0.596	0.668
**Edge of solid components in part-solid nodules**	0.226	0.202	-0.02	-0.034	0.556	0.633
**Interlobular septa**	0.148	0.234	-0.167	0	1	0.76
**Edge of vessels in ground-glass opacities**	0.161	0.183	-0.032	-0.098	0.488	0.741
**Edge of solid nodules**	0.204	0.092	-0.062	-0.072	0.625	0.667
**Interlobar fissures**	0.334	0.258	0.016	0	0.913	1
**Pleural indentations**	0.133	0.206	-0.154	-0.333	0.444	0.667

### CT-pathologic correlation of a part-solid nodule

**Fig 5:**
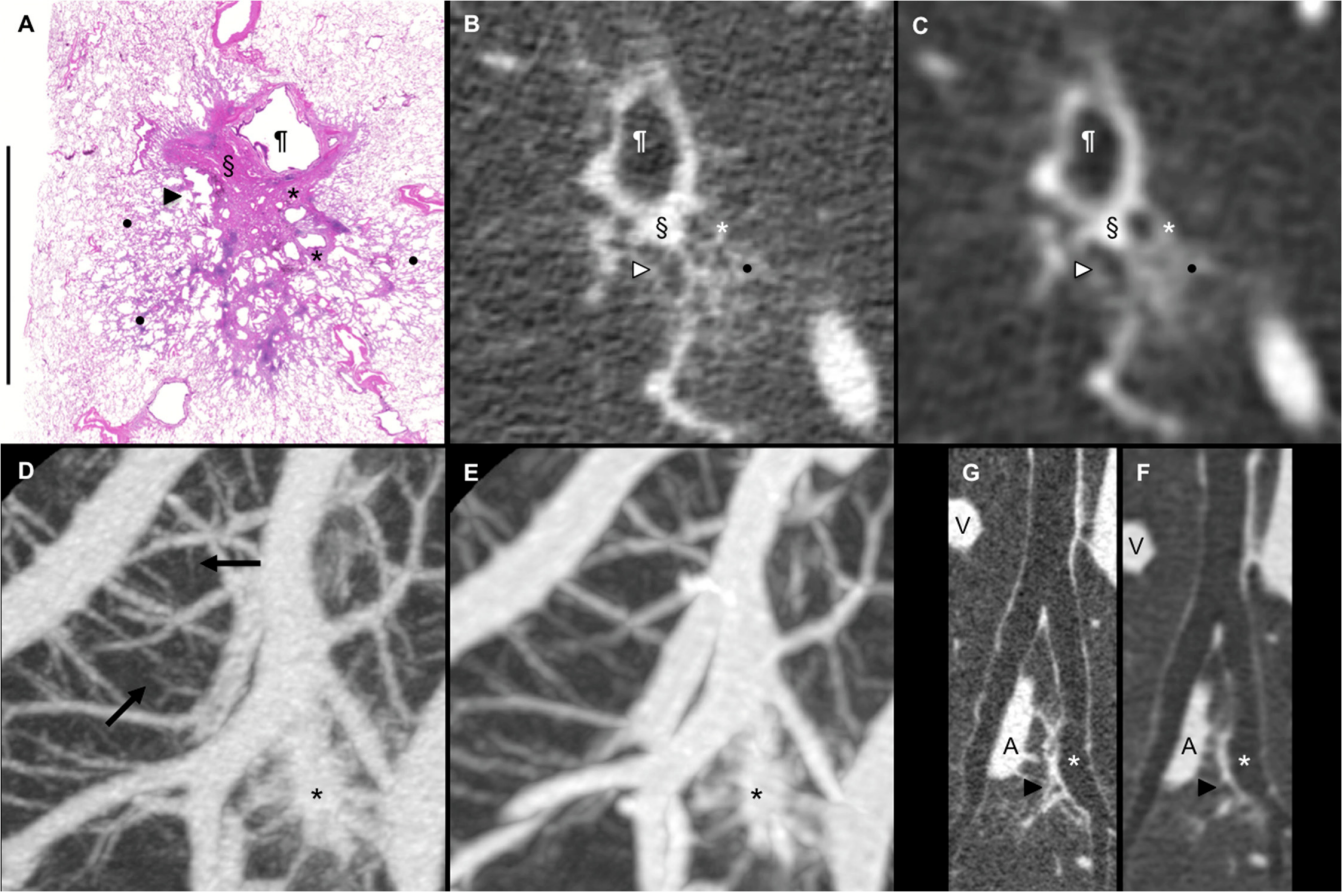
CT-pathologic correlation of an adenocarcinoma in situ. The patient was a 58-year-old female with adenocarcinoma in situ (size, 18 x 16 mm; pT1aN0M0, stage IA). The part-solid nodule was located in segment 6 of the right lower lobe. (A) Loupe view of the pathology specimen (Scale: 1 cm) (H & E, original magnification x1.25). (B) Multiplanar reconstruction (MPR) image from the ultra-high-resolution CT (U-HRCT) data corresponding to the pathology specimen. (C) MPR image from the conventional high-resolution CT (C-HRCT) data corresponding to the pathology specimen. U-HRCT image (Fig B) clearly depicting the solid component (§) immediately adjacent to the bronchial wall (¶) and low-attenuation areas, indicated by a white arrowhead and a white star, in the GGO component of the part-solid nodule. Collapse of the alveolar spaces (§) in Fig 5A corresponds to the solid component (§) of the part-solid nodule on the U-HRCT and C-HRCT images (Fig 5B and 5C), and the nlarged bronchiole (black arrowhead) and enlarged alveolar air spaces (black stars) in Fig 5A correspond to the low-attenuation areas (white arrowhead and white star) in the GGO component of the part-solid nodule on the U-HRCT and C-HRCT images (Fig 5B and 5C). The lepidic component indicated by the black dots in Fig 5A corresponds to the GGO component (black dot) of the part-solid nodule on the U-HRCT and C-HRCT images (Fig 5B and 5C). (D) Maximum intensity projection (MIP) image (2 cm thick) of the pulmonary vessels obtained from the U-HRCT data. (E) MIP image (4 cm thick) of the pulmonary vessels obtained from the C-HRCT data. Fine pulmonary vessels are depicted on the MIP image reconstructed from the U-HRCT data (black arrows). The size of the fine pulmonary vessels, as indicated by the black arrows (Fig 5D), was 0.2 mm. The black star in each MIP image indicates the part-solid nodule of the adenocarcinoma in situ. (F) 3D curved-MPR image of the pulmonary bronchi from the U-HRCT data. (G) 3D curved-MPR image of the pulmonary bronchi from the C-HRCT data. U-HRCT image (Fig 5F) clearly depicting the part-solid opacity (black arrowhead) immediately adjacent to the bronchial wall (white star) with traction bronchiectasis caused by the collapse of the alveoli in the tumor. A: pulmonary artery. V: pulmonary vein.

Fig 5 shows a loupe view of the pathological specimen of an adenocarcinoma in situ (Fig 5A), a corresponding MPR image obtained from the U-HRCT data (Fig 5B), and a corresponding MPR image obtained from the C-HRCT data (Fig 5C). The ectatic bronchiole and enlarged alveolar air spaces in the part-solid nodule are clearly visualized on the U-HRCT image. The pulmonary vessels are depicted more finely in the MIP image reconstructed from the U-HRCT data (Fig 5D) than in that reconstructed from the C-HRCT data (Fig 5E). The 3D curved-MPR image of the pulmonary bronchi obtained from the U-HRCT data (Fig 5F) shows the bronchial walls more sharply than the MPR image obtained from the C-HRCT data (Fig 5G).

## Discussion

The present study is, to the best of our knowledge, the first to evaluate the quality of imaging findings of lung nodules obtained using a prototype U-HRCT scanner. Although the U-HRCT images had more noise (i.e., the mean standard deviation of the CT values of the phantom was twice as great) than the C-HRCT images, their overall quality, as determined using slit phantoms, was superior, with a spatial resolution approximately 3 times as great. The greater image quality of U-HRCT might affect the management of both subsolid nodules, i.e., pure ground-glass nodules and part-solid nodules [21] and solid nodules [22].

Among subsolid nodules and solid nodules, part-solid nodules, which have both a ground-glass component and a solid component(s), have the greatest likelihood of malignancy [23]. For part-solid nodules, the quality of the U-HRCT images depicting the edge of the solid component was greater than that observed using the C-HRCT images (Tables 1 and 2). According to the International Association for the Study of Lung Cancer, the American Thoracic Society, and the European Respiratory Society’s proposed international multidisciplinary classification for lung adenocarcinoma, adenocarcinomas are classified as preinvasive lesions (i.e., atypical adenomatous hyperplasia [AAH] or adenocarcinoma in situ [AIS]), minimally invasive adenocarcinoma (MIA), or invasive adenocarcinoma [24]. One study of pathologic stage IA lung adenocarcinoma reported that the 5-year survival rates of AIS and MIA cases were 100%; in contrast, the 5-year survival rate of invasive adenocarcinoma cases was 78.4%; this difference was statistically significant (*P* < .01) [25]. Therefore, the differentiation of AIS and MIA from invasive adenocarcinomas is crucial. According to the international multidisciplinary classification of lung adenocarcinoma, AIS is defined as a small nodule (≤3 cm) exhibiting pure lepidic growth; MIA is defined as a small nodule (≤3 cm) exhibiting predominantly lepidic growth and ≤5 mm of invasion; and lepidic-predominant invasive nonmucinous adenocarcinoma (LPA), a subgroup of invasive adenocarcinomas, is defined as exhibiting lepidic growth and >5 mm of invasion [24]. On HRCT images, AIS is usually visualized as a pure ground-glass nodule, but may be part-solid or bubblelike; MIA is visualized as a part-solid nodule consisting of a predominant ground-glass component and a small solid component measuring 5 mm or less (this description has been proposed as an interim formulation); and LPA is usually visualized as a part-solid nodule, but may be pure ground-glass nodule or occasionally bubblelike [24, 26]. A key point for differentiating MIA from LPA on HRCT images is that MIA is defined as having a solid component not larger than 5 mm. Although the size of invasive foci on pathological specimens does not correspond to the size of solid components on HRCT images, an interim proposed threshold for differentiating MIAs from LPAs is a size of 5 mm [21, 26]; according to the Fleischner Society, if the solid component of a part-solid nodule is ≤5 mm in size, yearly surveillance CT examinations are recommended for a minimum of 3 years [21]. Future study is warranted regarding the visualization of solid components of part-solid nodules on U-HRCT images.

The differential diagnosis of solitary pulmonary solid nodules is based on several HRCT findings. Pleural indentation is a characteristic indicating a higher likelihood of malignancy not only for subsolid nodules [27], but also for solid nodules [28–30]. An air bronchogram is also useful for differentiating lung adenocarcinoma from benign solid nodules [4,31] and for predicting negative nodal involvement in solid lung cancer [32], whereas the interlobular septum is useful for differentiating intrapulmonary lymph nodes [33]. In the present study, the CT findings of pleural indentation, air bronchograms, and interlobular septa received higher scores on the U-HRCT images than on the C-HRCT images. Therefore, U-HRCT might prove useful for the differential diagnosis of solid nodules and the prediction of negative nodal involvement in solid cancers of the lung.

Some of the pairs of CT findings were missed and were not scored by the observers because the reading system for the CT images, which used differently colored dots with annotations and X and Y coordinates to indicate the findings, was overly complex. The reading system should have shown each CT finding one at a time, rather than showing several CT findings at once on each CT image. Moreover, to improve the reading system in future observer tests, if an observer has not completed the scoring of the CT findings on each CT image, the reading system should show the observer a dialogue box that says, “Some CT findings have not been scored.”

The interobserver agreement as assessed using κ statistics was poor or fair for each CT finding made using each CT scanner. Two main reasons may explain these results. First, for the 10 observers, this test was the first time that they had actually evaluated CT findings obtained using U-HRCT, despite having completed the training session before the observer test. Second, the 10 observers who participated in this observer test had different periods of experience reading chest CT images. One study reported poor interobserver agreement with evaluation of the multidetector CT image quality because of a difference between the experiences of radiologists 1 and 2 [17]. Another study showed that substantial variability remains across experienced thoracic radiologists in the task of lung nodule identification [34]. In the field of diagnostic imaging, inherent interobserver variability is unavoidable.

The CTDI_vol_ for the prototype U-HRCT findings obtained at 120 kV, 120 mAs or 135 kV, 105 mAs (CTDI_vol_ = 36.6 mGy [120 kV, 120 mAs], 39.1 mGy [135 kV, 105 mAs]) was greater than that for the C-HRCT findings obtained using a 64 detector-row multislice CT scanner at 120 kV, 150 mAs (CTDI_vol_ = 34.6 mGy). The reason for this difference was that the geometrical efficiency in the z-direction of a 4 detector-row multislice CT scanner (dose efficiency [DE] = 43%) is lower than that of recent multislice CT scanners, such as 16 detector-row multislice CT (DE = 70.2%) and 64 detector-row multislice CT scanners (DE = 80%). In the near future, as a larger number of detector-row U-HRCT scanners with improved geometrical efficiency in the z-direction become available, the radiation doses will be reduced.

The reason why two C-HRCT scanners were used to compare the different properties of the prototype U-HRCT scanner was that the subject accrual required a long period of time, i.e., 2 and a half years. During that time, the C-HRCT scanner changed from a 16-raw scanner to a 64-row scanner.

Regarding the difference in scan coverage between the C-HRCT scanners and the prototype U-HRCT scanner, the 4-cm coverage of the thorax using 16-row or 64-row C-HRCT was a routine procedure performed in the follow-up clinic at the Research Center for Cancer Prevention and Screening. For the prototype U-HRCT scanner, to utilize ultra-high resolution scan mode (beam collimation of 0.25 mm x 4, 0.75 seconds per rotation, pitch factor of 0.625), the scan coverage had to be restricted because of breath hold limitations. As a result, the scan range was set at 2 cm.

Micro-vessel imaging for pathological angiogenesis using micro-CT has been reported [35]; the study showed that the combination of functional in vivo and anatomical ex vivo micro-CT allows highly accurate quantification of the relative blood volume and highly detailed three-dimensional analysis of the vascular network in the tumors in mice; blood vessels as small as 3.4 μm in diameter could be visualized using ultra-high-resolution ex vivo micro-CT. Another in vivo micro-CT study reported that the mean diameter of the narrowed common carotid artery in mice was 0.253 mm [36]. Our study did not evaluate depiction of the pulmonary vessels based on the sizes of the vessels on the U-HRCT images and C-HRCT images; Fig 5D shows only the preliminary measurement results of the fine pulmonary vessels. Furthermore, we did not perform U-HRCT imaging using contrast. Therefore, further studies of micro-vessel imaging are warranted.

The present study had several limitations. First, we were not able to evaluate the ability of the prototype U-HRCT scanner to differentiate malignant nodules from benign nodules because approximately two-thirds of the patients had resected adenocarcinomas and the remaining one-third of patients had clinically benign or indeterminate nodules. However, this ability of the prototype U-HRCT scanner should be evaluated, and for this purpose we are now collecting cases of solitary pulmonary nodules. Second, the image noise for the prototype U-HRCT scanner was greater than that for the C-HRCT scanner. The size of the detector can be expected to affect the image noise because the area (0.25 x 0.25 mm^2^) of the detector in the U-HRCT was one-fourth the size of the area (0.5 x 0.5 mm^2^) of the detector in the C-HRCT. One solution would be to develop an optimal iterative reconstruction algorithm that could improve the quality of the CT images. A third limitation was that the gantry rotation speed of the prototype U-HRCT scanner was 0.75 seconds per rotation. Therefore, the image quality might have been affected by heartbeat or inadequate breath holding. If scanners with a faster gantry rotation are developed, the image quality might improve. Fourth, the maximal output of the tube current for the prototype U-HRCT scanner was smaller than that for the C-HRCT scanners; consequently, for larger patients, the image quality obtained using the prototype U-HRCT scanner was inferior to that obtained using the C-HRCT scanners. In the future, a higher maximal output of the tube current should be realized, even if for only a small focal spot. Finally, 1-mm beam collimation of the U-HRCT could not cover the whole thorax. In the future, U-HRCT scanners with a larger number of detector rows could cover the whole thorax in a short scanning time.

## Conclusion

In conclusion, the U-HRCT prototype scanner, despite having greater image noise, provides a significantly better image quality than C-HRCT scanners. The U-HRCT scanner might be useful for differentiating malignant pulmonary nodules from benign pulmonary nodules during workup or during follow-up of suspicious nodules detected using low-dose CT lung cancer screening.

## Supporting Information

S1 Table Dataset of Scores. (XLSX)
