# Correction: Ultra-High-Resolution Computed Tomography of the Lung: Image Quality of a Prototype Scanner

**DOI:** 10.1371/journal.pone.0145357

**Published:** 2015-12-18

**Authors:** Ryutaro Kakinuma, Noriyuki Moriyama, Yukio Muramatsu, Shiho Gomi, Masahiro Suzuki, Hirobumi Nagasawa, Masahiko Kusumoto, Tomohiko Aso, Yoshihisa Muramatsu, Takaaki Tsuchida, Koji Tsuta, Akiko Miyagi Maeshima, Naobumi Tochigi, Shun-ichi Watanabe, Naoki Sugihara, Shinsuke Tsukagoshi, Yasuo Saito, Masahiro Kazama, Kazuto Ashizawa, Kazuo Awai, Osamu Honda, Hiroyuki Ishikawa, Naoya Koizumi, Daisuke Komoto, Hiroshi Moriya, Seitaro Oda, Yasuji Oshiro, Masahiro Yanagawa, Noriyuki Tomiyama, Hisao Asamura

Fig 5F and 5G are improperly labelled. Please see the correct Fig 5 here.

**Fig 5:**
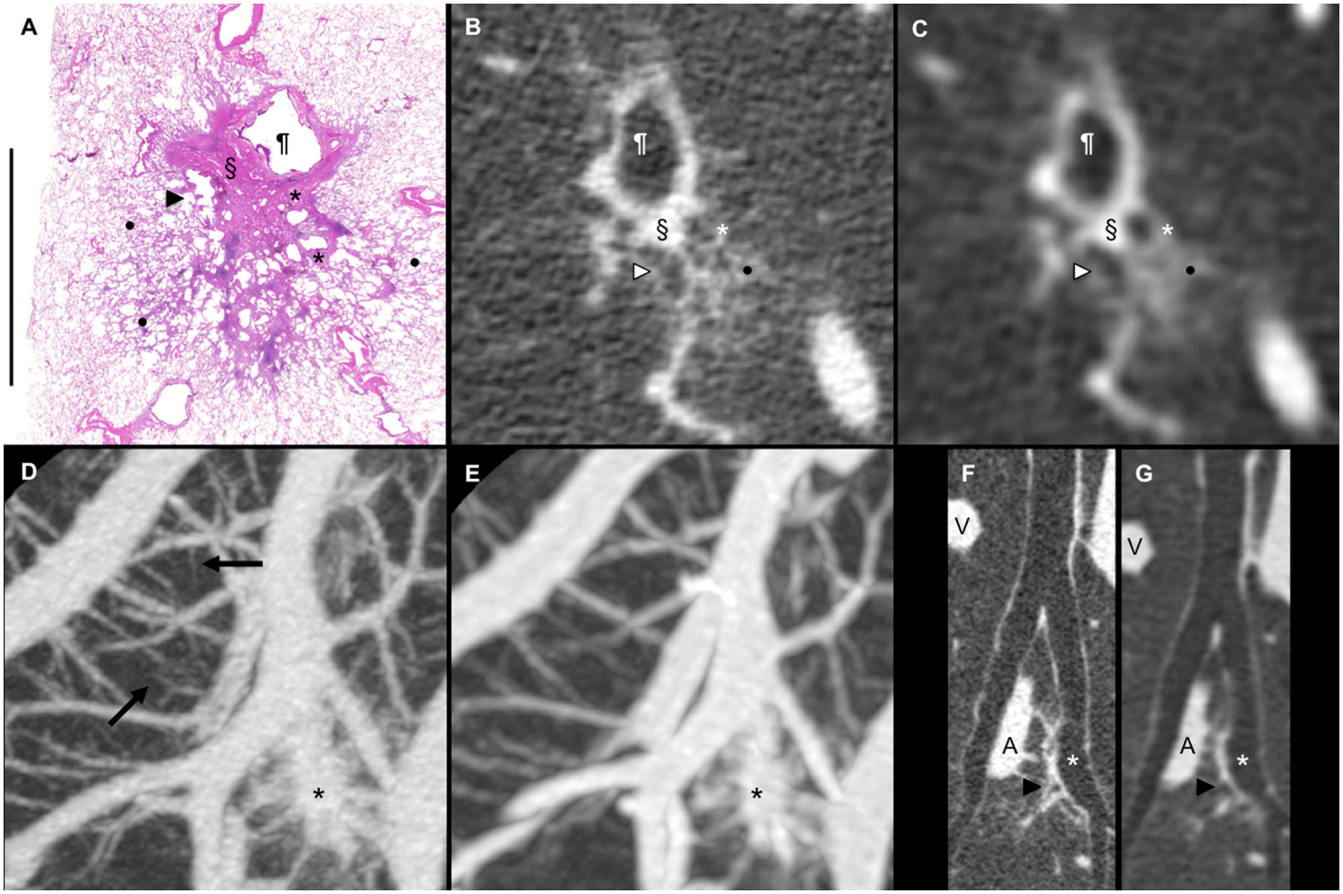
CT-pathologic correlation of an adenocarcinoma in situ. The patient was a 58-year-old female with adenocarcinoma in situ (size, 18 x 16 mm; pT1aN0M0, stage IA). The part-solid nodule was located in segment 6 of the right lower lobe. (A) Loupe view of the pathology specimen (Scale: 1 cm) (H & E, original magnification x1.25). (B) Multiplanar reconstruction (MPR) image from the ultra-high-resolution CT (U-HRCT) data corresponding to the pathology specimen. (C) MPR image from the conventional high-resolution CT (C-HRCT) data corresponding to the pathology specimen. U-HRCT image (Fig B) clearly depicting the solid component (§) immediately adjacent to the bronchial wall (¶) and low-attenuation areas, indicated by a white arrowhead and a white star, in the GGO component of the part-solid nodule. Collapse of the alveolar spaces (§) in Fig 5A corresponds to the solid component (§) of the part-solid nodule on the U-HRCT and C-HRCT images (Fig 5B and 5C), and the enlarged bronchiole (black arrowhead) and enlarged alveolar air spaces (black stars) in Fig 5A correspond to the low-attenuation areas (white arrowhead and white star) in the GGO component of the part-solid nodule on the U-HRCT and C-HRCT images (Fig 5B and 5C). The lepidic component indicated by the black dots in Fig 5A corresponds to the GGO component (black dot) of the part-solid nodule on the U-HRCT and C-HRCT images (Fig 5B and 5C). (D) Maximum intensity projection (MIP) image (2 cm thick) of the pulmonary vessels obtained from the U-HRCT data. (E) MIP image (4 cm thick) of the pulmonary vessels obtained from the C-HRCT data. Fine pulmonary vessels are depicted on the MIP image reconstructed from the U-HRCT data (black arrows). The size of the fine pulmonary vessels, as indicated by the black arrows (Fig 5D), was 0.2 mm. The black star in each MIP image indicates the part-solid nodule of the adenocarcinoma in situ. (F) 3D curved-MPR image of the pulmonary bronchi from the U-HRCT data. (G) 3D curved-MPR image of the pulmonary bronchi from the C-HRCT data. U-HRCT image (Fig 5F) clearly depicting the part-solid opacity (black arrowhead) immediately adjacent to the bronchial wall (white star) with traction bronchiectasis caused by the collapse of the alveoli in the tumor. A: pulmonary artery. V: pulmonary vein.
